# Relationship Between Toe Clearance Strategy and Regional Regulation of Rectus Femoris Muscle During Swing Phase in Prolonged Walking in Young and Older Adults

**DOI:** 10.3389/fphys.2018.01274

**Published:** 2018-09-06

**Authors:** Kohei Watanabe

**Affiliations:** Laboratory of Neuromuscular Biomechanics, School of International Liberal Studies, Chukyo University, Nagoya, Japan

**Keywords:** neuromuscular compartment, bi-articular muscles, multi-channel surface electromyography, aging, tripping

## Abstract

The toe clearance strategy during leg swinging while walking is closely associated with the risk of tripping and/or falling and is influenced by aging and a fall history. However, it remains unclear how the toe clearance strategy is regulated by the neuromuscular system. The present study investigated the effect of aging and fall/tripping history in the older adults on the toe clearance strategy and neuromuscular regulation of the rectus femoris (RF) muscle, which plays an important role in leg swinging, during prolonged walking. Thirteen older adults (age: 71.3 ± 5.7 years) and nine young adults (age: 20.9 ± 0.8 years) men volunteered for the present study. The older adults were divided into those with (*n* = 6) and without (*n* = 7) a fall/tripping history. Subjects walked on a treadmill at their preferred gait speed for 20 min, and lower extremity kinematics and multi-channel surface electromyography along the RF muscle were recorded. Variability of the minimum toe clearance (MTC) and central locus activation (CLA) of the RF muscle in older adults was significantly greater than in the young adults (*p* < 0.05). MTC significantly decreased with time in the older adults (*p* < 0.05), but not in the young adults (*p* > 0.05). There were no significant correlations between any parameters of MTC and CLA in the older adults or young adults (*p* > 0.05). MTC and variability of CLA significantly decreased with time in the older adults without a fall/tripping history (*p* < 0.05), but not in the older adults with such a history (*p* > 0.05). These results suggest that aging and a fall/tripping history in the older adults alter the toe clearance strategy and regional neural regulation of the RF muscle during prolonged walking.

## Introduction

The risk of trip-related falls is maximal when the distance between the foot of the swing leg and the surface being walked on, i.e., minimum foot clearance (MFC), is reduced to zero or very small (Winter, 1992; Barrett et al., 2010). Variability of MFC during repeated gait cycles increases with aging and the presence of a fall history, and is known as a risk indicator for trips and related falls in the older adults (Karst et al., 1999; Mills and Barrett, 2001; Begg et al., 2007; Mills et al., 2008; Barrett et al., 2010). While Mills et al. (2008) reported detailed relationships between lower extremity kinematics and MFC during gait in the young and older adults (Mills et al., 2008), the relationship with neuromuscular regulation is not fully understood.

It is well known that the rectus femoris (RF) muscle is closely associated with the gait function, and some pathological gait patterns are caused by abnormality of RF muscle activity (Sung et al., 2003; Chantraine et al., 2005; Reinbolt et al., 2008). We previously reported the unique nature of neuromuscular regulation in the RF muscle during gait. While the whole RF muscle is activated during the stance phase, its proximal region is selectively recruited during the swing phase (Watanabe et al., 2014b). This phenomenon can be explained by the region-specific functional role of the RF muscle, i.e., the proximal region of the RF muscle preferentially contributes to the hip flexion joint moment (Hagio et al., 2012; Watanabe et al., 2012, 2014a). Since the RF muscle contributes to two different joint moments, knee extension and hip flexion, regional neuromuscular control of the proximal RF muscle plays a role in minimizing unexpected knee extension joint moment induced by RF muscle activation during the swing phase. On the other hand, we also noted that this regional regulation of the RF muscle during the swing phase of gait is affected by aging. In the older adults, additional activation at distal region of the RF muscle was observed with activation at proximal regions during the swing phase (Watanabe et al., 2016). This may lead to unexpected knee extension joint moment during the swing phase and alteration in the toe clearance strategy.

The gait pattern and toe clearance strategy are modified during prolonged walking. Yoshino et al. (2004) reported that prolonged walking leads an increase in the gait cycle and its variability and mediolateral acceleration of the center of gravity (Yoshino et al., 2004). Nagano et al. (2014) showed that MTC is reduced due to prolonged walking-induced fatigue in the older adults, but not in young adults (Nagano et al., 2014). On the other hand, Barbieri et al. (2014) found that the effect of fatigue on heel clearance pattern during obstacle avoidance was not directly related with age (Barbieri et al., 2014). There are differences in the findings for relationship between age and foot clearance strategy during walking with fatigue. Also, older adults with a history of trip-related falls show a unique toe clearance strategy (Khandoker et al., 2008a,b), higher position of and greater variability of MFC (Barrett et al., 2010). However, no study has investigated the relationship between neuromuscular regulation and the toe clearance strategy during prolonged walking or in the older adults with a fall history.

The present study aimed to clarify the effect of aging and a fall/tripping history in the older adults on the toe clearance strategy and neuromuscular regulation of the RF muscle during prolonged walking. The following hypothesis were tested during prolonged walking: 1) MFC variability is associated with variability of regional activation of the RF muscle in the older adults, and 2) older adults with a fall/tripping history show a unique toe clearance strategy and neuromuscular regulation of the RF muscle.

## Materials and Methods

### Subjects

Thirteen older adults (age: 71.3 ± 5.7 years, height: 167.3 ± 5.1 cm, body mass: 62.6 ± 7.1 kg) and nine young adults (age: 20.9 ± 0.8 years, height: 174.0 ± 6.7 cm, body mass: 63.0 ± 6.6 kg) men volunteered for the present study. Since women generally have thicker subcutaneous fat tissue that decreases surface EMG signal and increases signal-noise ratio in surface EMG signal, only men were chosen as the subjects in this study. The subjects gave written informed consent for the study after receiving a detailed explanation of the purposes, potential benefits, and risks associated with participation. All subjects were healthy with no history of any musculoskeletal or neurological disorders. All study procedures were conducted in accordance with the Declaration of Helsinki and research code of ethics of Chukyo University and were approved by the Committee for Human Experimentation of Chukyo University (2014-001 and 2017-004).

### Experimental Design

Subjects were familiarized with walking on treadmill for 6∼16 days before the experimental day. On a trial day, the preferred gait speed was measured while walking a distance of 10 m with a normal gait along a flat surface. On the experimental day, the subjects walked on a treadmill (MEDTRACK ST65, Quinton Instrument Co., WA, United States) at their preferred gait speed for 20 min and lower extremity kinematics and neuromuscular activation of the RF muscle were recorded. The right leg was used to analyze joint kinematics and neuromuscular activation in this study. We directly questioned the trip history including fall and dragging for the older adults. The present study set that the elderly with fall/tripping history was who experienced a tripping (dragging) lately or a falling within a year.

### Lower Extremity Kinematics

**FIGURE 1:**
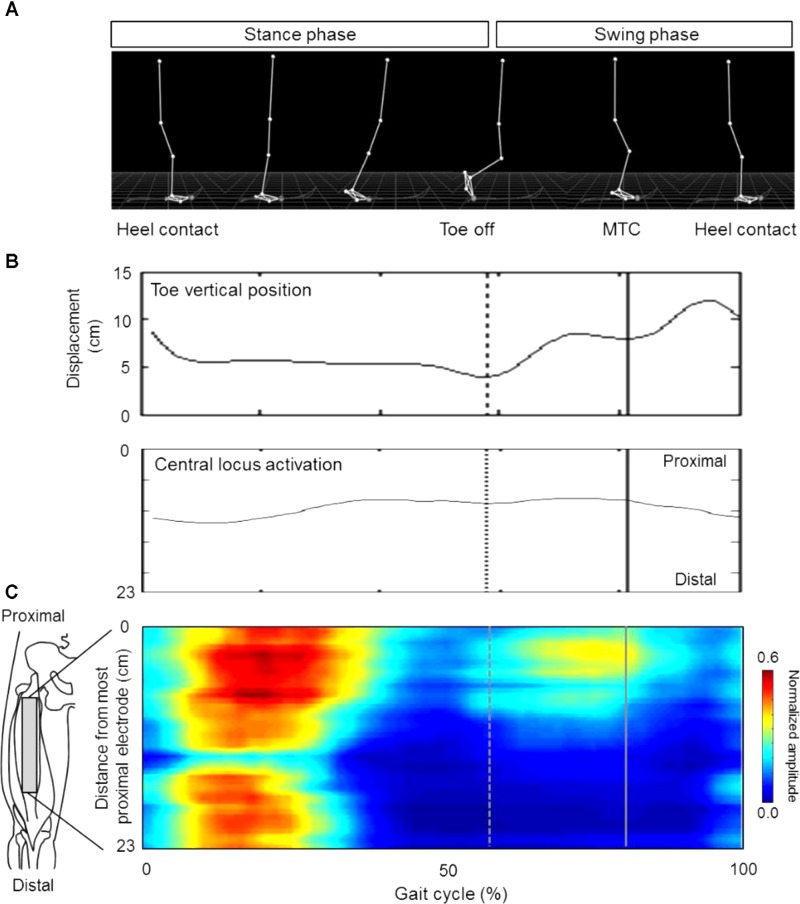
Representative data on the lower joint kinematics **(A)**, toe vertical position **(B)** and normalized multi-channel surface electromyography amplitude of the rectus femoris muscle **(C)** for calculating minimum toe clearance (MTC) and central locus activation (CLA). Vertical solid and broken lines indicate the timings of MTC and toe off, respectively.

Coordinates on the sagittal plane obtained by placing reflective markers on the lower extremities were obtained using a three-dimensional motion capture system with six cameras (Vicon Bonita 3, Vicon Motion Systems Ltd., Oxford, United Kingdom) at a sampling rate of 100 Hz. While reflective markers were attached to the right acromion, greater trochanter, lateral femoral epicondyle, lateral malleolus, fifth metatarsal bone, toe, and heel, the markers on the toe and heel were used to determine heel contact and toe-off and analyze the toe clearance trajectory. To identify heel contact and toe off timings, vertical coordinates of heel and toe were measured during static standing before prolonged walking. The timings of heel contact or toe off were defined as beginning of stance or swing phases when vertical coordinate of heel or toe was lesser or greater than the vertical coordinates at the static standing. The detected coordinates on the sagittal plane for each marker were filtered with a fourth order Butterworth low-pass filter (6 Hz). To assess the individual characteristics of toe clearance, we calculated the MFC for each stride, i.e., lowest displacement between two highest peaks of vertical toe position from toe-off to heel contact (swing phase) (**Figure 1**). Toe clearance strategy was assessed by this parameter. MFC values from 5 to 10 min and 15 to 20 min were used for further analysis. To synchronize the motion capture and EMG data, infrared radiation light-emitting diode with electrical signals were used.

### Surface EMG Recording

Neuromuscular activation of the RF muscle was assessed by multi-channel surface EMG. Since regional EMG responses along the longitudinal axis of the RF muscle were noted in a previous studies (Watanabe et al., 2012, 2014b), 24 electrodes, with a 6 × 4 electrode arrangement (1 × 5 mm detection area, 10-mm inter-electrode distance; ELSH004, OT Bioelectronica, Turin, Italy) were attached along the line between the anterior superior iliac spine and superior edge of the patella (Watanabe et al., 2016). Surface EMG signals were recorded at 2,048 Hz with an eighth order Bessel band pass filter at 10–750 Hz (anti-aliasing filter) (EMG-USB, OT Bioelectronica, Torino, Italy). Bipolar surface EMG signals were calculated from the electrode pairs between neighboring electrodes along the rows in each array, rectified, and normalized by the peak value for each channel in each stride. Using the eighteen EMG signals from 3 pairs × 6 array electrodes along the longitudinal axis of the RF muscle, the centroid of the normalized rectified EMG along the muscle, i.e., central locus activation (CLA), was calculated in inter-electrode distance units at the time of MFC for each stride. This variable reflects regional neuromuscular regulation of the RF muscle. The results for CLA are shown as the distance (cm) from the most proximal edge of electrodes (**Figure 1**). CLA values from 5 to 10 min and 15 to 20 min were used for further analysis and to test the relationship with MFC.

### Statistics

In the present study, non-parametric tests were used since the sample size was small and data distribution was partly non-gaussian. MFC, CLA, and gait parameters such as cadence, toe off timing, and timing of MFC were compared between the older and young groups using the Mann-Whitney test to clarify the effect of aging and compared between 5–10 and 15–20 min using the Wilcoxon rank sum test to determine the effect of time for each subject group. Also, these variables were compared between the older adults with and without fall/tripping history using the Mann-Whitney test. Spearman’s rank correlation coefficient was calculated between MFC and CLA at 5–10 min and 15–20 min to test the association between toe clearance and regional neuromuscular regulation of the RF muscle. The level of significance was set at *p* < 0.05. Statistical analysis was performed using SPSS (version 15.0, SPSS, Tokyo, Japan) and MATLAB (MATLAB R2008a, MathWorks, MA, United States).

## Results

**FIGURE 2:**
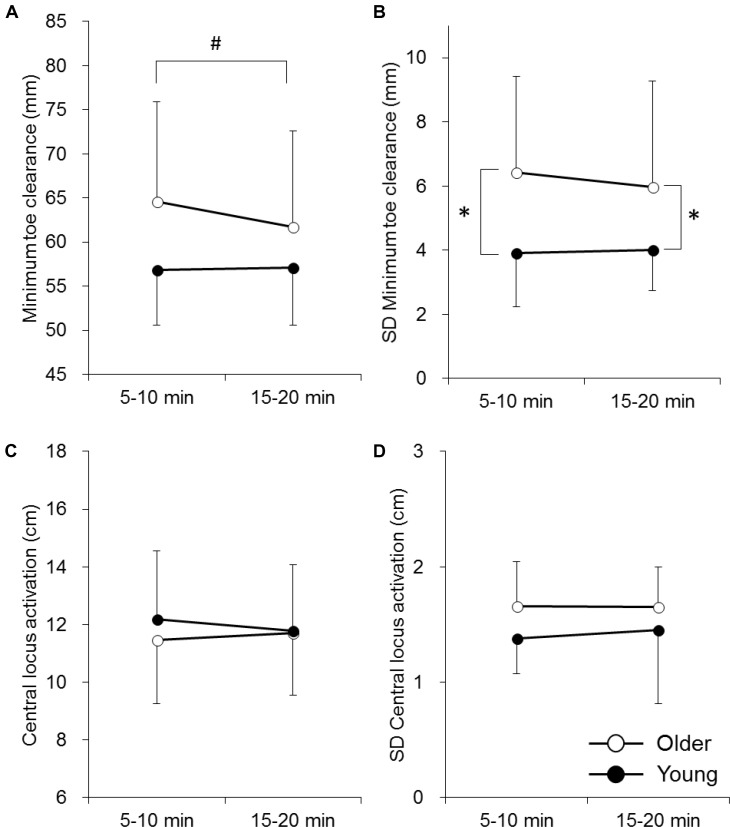
Mean and standard deviation of minimum toe clearance (MTC) **(A,B)** and central locus activation (CLA) of the rectus femoris muscle **(C,D)** in the older adults (White circle) and young adults (Black circle). The symbols ∗ and # indicate significant differences (*p* < 0.05) between the older adults and young adults and between 5–10 min and 15–20 min, respectively.

Gait parameters used in this study are shown in **Table 1**. There were no significant differences in these gait parameters between the older and young adults (*p* > 0.05) (**Table 1**). Cadence in the older adults significantly decreased with time (*p* < 0.05), but no other parameters changed over time in either group (*p* > 0.05) (**Table 1**). There were no significant differences in the mean MTC or CLA at 5–10 min and 15–20 min between the older and young adults (*p* > 0.05) (**Figures 1A,C**). There were significant differences in SD of MTC at 5–10 min and 15–20 min between the older and young adults (*p* < 0.05) (**Figure 2B**). In the older adults, a significant effect of time was found in mean of MTC (*p* < 0.05) (**Figure 2A**). No significant correlations were found between parameters of MTC and CLA at 15–20 min in the older and young adults (*p* > 0.05) (**Table 2**).

**Table 1:** Gait parameters in older and young groups.

		Older adults	Young adults
		*N* = 13	*N* = 9
Preferred gait speed (km/h)		4.7 ± 0.7	5.2 ± 0.7
Sample step number	5–10 min	289.1 ± 25.3	290.0 ± 15.5
	15–20 min	289.6 ± 16.1	291.0 ± 13.0
Cadence (bpm)	5–10 min	118.9 ± 8.1	119.3 ± 7.9
	15–20 min	116.8 ± 8.3^∗^	117.8 ± 6.5
Toe off (% of stride)	5–10 min	62.1 ± 2.1	61.1 ± 2.4
	15–20 min	62.2 ± 1.9	61.7 ± 1.7
Time of MFC (% of stride)	5–10 min	83.3 ± 2.2	82.8 ± 0.8
	15–20 min	83.1 ± 2.0	83.0 ± 0.8


*^∗^*p* < 0.05 vs. 5–10 min.*

**Table 2:** Correlation coefficients between minimum toe clearance and central locus activation.

		Older adults	Young adults
		r	r
Mean	5–10 min	0.209	-0.417
	15–20 min	0.418	-0.517
*SD*	5–10 min	0.148	0.200
	15–20 min	0.258	0.000


**FIGURE 3:**
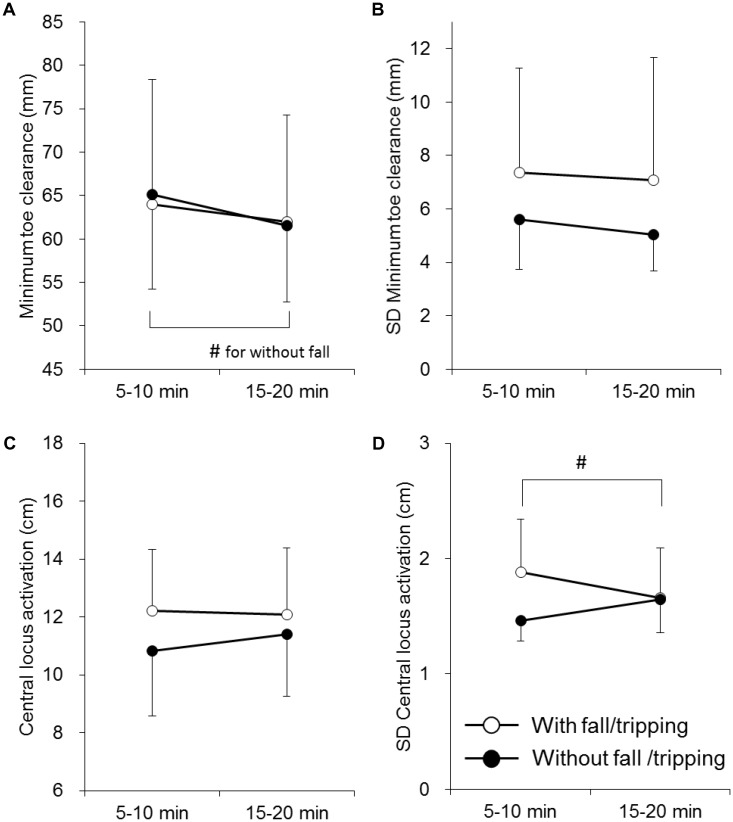
Mean and standard deviation of minimum toe clearance (MTC) **(A,B)** and central locus activation (CLA) of the rectus femoris muscle **(C,D)** in the older adults with (White circle) and without (Black circle) fall/tripping history. The symbol # indicates a significant difference (*p* < 0.05) between 5—10 min and 15–20 min.

There were no significant differences in gait parameters, MTC, or CLA between the older adults with and without fall/tripping history (*p* > 0.05) (**Table 3** and **Figure 3**). A significant effect of time on the mean MTC was noted in the older adults without a fall/tripping history (*p* < 0.05), but not in the older adults with such a history (*p* > 0.05) (**Figure 3A**). SD of CLA was significantly influenced by time in the older adults with fall/tripping history (*p* < 0.05) (**Figure 3D**). There were significant positive correlations between mean values of MTC and CLA at 5–10 min and 15–20 min in the older adults with fall/tripping history (*p* < 0.05), but not in the older adults without fall/tripping history (*p* > 0.05) (**Table 4**).

**Table 3:** Profiles and gait parameters in the older adults with and without fall/tripping history.

		Older adults with fall/tripping history	Older adults without fall/tripping history
		*N* = 6	*N* = 7
Age (years)		69.0 ± 3.7	73.3 ± 6.5
Height (cm)		164.6 ± 5.6	169.6 ± 3.5
Body mass (kg)		59.8 ± 4.6	65.0 ± 8.2
Preferred gait speed (km/h)		4.6 ± 0.9	4.8 ± 0.5
Sample step number	5–10 min	283.3 ± 22.6	294.0 ± 28.3
	15–20 min	286.7 ± 19.0	292.1 ± 14.2
Cadence (bpm)	5–10 min	118.1 ± 10.7	119.6 ± 6.0
	15–20 min	116.2 ± 10.4	117.3 ± 7.0
Toe off (% of stride)	5–10 min	63.2 ± 1.4	61.1 ± 2.2
	15–20 min	63.3 ± 1.6	61.2 ± 1.7
Time of MFC (% of stride)	5–10 min	84.3 ± 1.4	82.4 ± 2.4
	15–20 min	84.0 ± 1.6	82.3 ± 2.0


**Table 4:** Correlation coefficients between minimum toe clearance and central locus activation for older adults with and without fall/tripping history.

		With fall/tripping history	Without fall/tripping history
		r	r
Mean	5–10 min	0.829^∗^	0.036
	15–20 min	0.829^∗^	0.143
*SD*	5–10 min	0.486	-0.286
	15–20 min	0.257	0.464


*^∗^*p* < 0.05.*

## Discussion

The present study investigated the effect of aging and a fall/tripping history in the older adults on the toe clearance strategy and neuromuscular regulation of the RF muscle during prolonged walking. The main findings of the present study were (1) variability of MTC in the older adults was significantly greater than in the young adults (**Figure 2B**), (2) MTC significantly decreased with time in the older adults, but not the in young adults (**Figure 2A**), (3) there were no significant correlations between parameters of MTC and CLA in the young or older adults (**Table 2**), and (4) the effects of time on the mean MTC and variability of CLA were not uniform between the older adults with and without a fall/tripping history (**Figures 3A,D**). These results do not support the first hypothesis of this study that MFC variability is associated with variability in regional activation of the RF muscle in older adults. On the other hand, the second hypothesis that older adults with a fall/tripping history show a unique toe clearance strategy and neuromuscular regulation of the RF muscle was supported by our results.

As reported in a previous study (Barrett et al., 2010), while the mean MTC was not different between the older and young adults (**Figure 1A**), SD of MTC, which is a parameter of variability, was significantly higher in the older adults (**Figure 1B**). In their review, Barrett et al. (2010) suggested that mechanisms of greater MTC variability in the older adults are unclear, but it may be induced by age-related reductions in proprioceptive sensitivity and their ability to modulate muscle force (Barrett et al., 2010). The present study investigated the latter possibilities by assessing regional neuromuscular regulation of the RF muscle. In our previous study, CLA in the older adults was located at a more distal site compared with the young adults during 70–80% of the gait cycle (Watanabe et al., 2016), revealing that preferential activation of the RF muscle was attenuated during the swing phase in the older adults. We considered that this unique activation pattern in the older adults would lead to unexpected knee extension and a lower toe position during the swing phase. However, there were no significant differences in CLA between the older and young adults in the present study (*p* > 0.05) (**Figure 2** and **Table 2**). This may be partly due to differences in the gait speed, subjects’ profiles, and analyzed phase between the present and previous studies. While age-related differences in CLA were noted solely with a fast gait speed and only the older adults without a fall history participated in a previous study (Watanabe et al., 2016), the present study employed a normal gait speed and the older adults subjects included those with fall/tripping history. The previous and present studies compared CLA between older and young adults at 70–80% (Watanabe et al., 2016) and approximately 83% of stride, respectively. There were no significant differences in CLA at 80–90% of stride for any gait speeds in the previous study (Watanabe et al., 2016). One of the causes of difference in the results between previous and present study could be the analyzed phase. On the other hand, the present study also investigated the effect of prolonged walking on CLA. Although the gait pattern in the older adults was affected by prolonged walking, i.e., decreases in cadence and MTC (**Table 1** and **Figure 2A**), CLA remained unchanged between 5–10 min and 15–20 min in the older adults (**Figure 2C**). Moreover, correlations between mean values and variabilities in MTC and CLA were not detected in either the older or young adults subjects (**Table 2**). These findings suggest that the age-related toe clearance strategy was not associated with the regional neuromuscular activation pattern in the RF muscle. This is the answer to major question in the present study. During gait, large number of the muscles including the RF muscle are recruited and other muscles such as tibialis anterior or triceps surae muscles would have greater contribution to toe clearance strategy. It would be difficult to explain toe clearance strategy by only the RF muscle and combination of recording surface EMG from other key muscles provide further understanding of toe clearance strategy and its aging.

In the present study, the effect of fall/tripping history on the toe clearance strategy and regional neuromuscular activation pattern in the RF muscle was investigated. First, it must be noted that this study combined histories of fall and tripping while these are clearly different events. Fall history directly affect motor behavior due to anxiety and/or fear (Young and Mark Williams, 2015), however, this could not be presented by tripping history. Therefore, comparison of the results in this study with those in the previous studies using the subjects with fall history only would be needed to pay attention. There were no significant differences in the mean or variability of MTC between the older adults with and without fall/tripping history (*p* > 0.05) (**Figures 3A,B**). In previous studies, greater mean values and variabilities in the older adults with a fall/tripping history were reported, and it was suggested that the change in the toe position may be a strategy to reduce the risk of falls associated with greater MTC variability (Khandoker et al., 2008a,b; Barrett et al., 2010). Although differences in the mean and variability of MTC were not observed in the present study, a difference in the time course of MTC during prolonged walking was noted on comparing the older adults with and without fall/tripping history (**Figure 3A**). MTC was significantly decreased with time in the older adults without fall/tripping history (*p* < 0.05), but not in the older adults with such history (*p* > 0.05) (**Figure 3A**). Considering the reduced MTC after prolonged walking in all older adults in this study (**Figure 2A**) and normal older adults in a previous study (Nagano et al., 2014), the unchanging MTC over time could be a characteristic toe clearance strategy in the older adults with a fall/tripping history during prolonged walking (**Figure 3A**). While reduction of the vertical toe position during the swing phase would increase the fall risk, maintaining a higher toe position during prolonged walking may increase the metabolic cost (Barrett et al., 2010). We think that the older adults with and without fall/tripping history adopt strategies that minimize the fall risk and metabolic energy, respectively. The present study also showed a difference in regional neuromuscular activation of the RF muscle between the older adults with and without a fall/tripping history. A significant decrease with time in variability in CLA was observed only in the older adults with a fall/tripping history (**Figure 3D**). While it is difficult to link this with the results regarding the toe clearance strategy, this neural activation pattern may be characteristic of the older adults with fall/tripping history. CLA is determined by the spatial distribution of the neuromuscular activation levels in proximal to distal regions of the RF muscle. Our previous studies already revealed that the regional neuromuscular activation pattern along the RF muscle is modified by motor tasks: knee extension or hip flexion (Watanabe et al., 2012), fatigue (Watanabe et al., 2013), and the joint angle (Watanabe et al., 2014a). From the results of these previous studies, the regional neural regulation of the RF muscle is modulated and integrated by many factors. Although the physiological mechanisms are unclear, decreased CLA variability over time in the older adults with a fall/tripping history could be due to reduced variability of these modulations. Significant correlation was found between mean values of MTC and CLA in the older adults with fall/tripping history at 5–10 min and 15–20 min (*p* < 0.05) (**Table 4**). This relationship was not observed in the analysis for all older adults (*p* > 0.05) (**Table 2**) and the older adults without fall/tripping history (*p* > 0.05) (**Table 4**). Positive relationship between MTC and CLA means that higher toe vertical position in the subjects with more distal position of CLA. This relationship is not reasonable, because our previous studies suggested that neuromuscular activation at proximal regions of the RF muscle contributes to hip flexion joint torque that should lead higher vertical toe position (Watanabe et al., 2012, 2014b, 2016). While it is difficult to clarify physiological/biomechanical mechanisms of this characteristic relationship between MTC and CLA in the older adults with fall/tripping history in this study, this may reflect unique gait strategy in the older adults with fall/tripping history. Also, it should be noted that the present study investigated the effect of fall/tripping history by including subjects with such a history and history of tripping and dragging, while the previous studies only included older subjects with a fall history. Differences in results between the present and previous studies may be partly explained by variations in subjects’ gait ability. The subjects with fall/tripping history in this study may be categorized as showing milder gait dysfunction when compared with those of previous studies (Khandoker et al., 2008a,b). On the other hand, I must note that the results in the present study were observed from small number of the subjects. This could be one of the limitations and interpretation of the results is also restricted in current study.

The present study focused on only the RF muscle, since this study performed to clarify the hypothesis that regional neuromuscular activation within the RF muscle is associated with gait parameters. However, many muscles are recruited during gait and the muscles around ankle joint would strongly contribute to toe clearance strategy. The further study that covers key muscles with the RF muscle could be needed to understand the effect of aging and fall/tripping history on the toe clearance strategy. Also, this study analyzed neuromuscular activation of the RF muscle and toe clearance strategy only in right leg. From the older adults may have asymmetry in the leg movements during walking, analyses of both legs could be needed for further understanding.

## Conclusion

The present study investigated the effect of aging and fall/tripping history in the older adults on the toe clearance strategy and neuromuscular regulation of the RF muscle during prolonged walking. To achieve this, we compared indicators of the toe clearance strategy and regional muscle activation along the RF muscle: MTC and CLA, between the older and young adults and between the older adults with and without a fall/tripping history during 20 min walking at normal speed. We noted a difference in MTC between the older and young adults, but not in CLA, and a difference in MTC and CLA during prolonged walking between the older adults with and without a fall/tripping history. Also, there were no significant correlations between parameters of MTC and CLA in the older adults. These results suggest that aging and a fall/tripping history in the older adults alter the toe clearance strategy and regional neural regulation of the RF muscle during prolonged walking, but toe clearance strategy and regional neural regulation of the RF muscle are not associated in the older adults.

## Author Contributions

The author confirms being the sole contributor of this work and approved it for publication.

## Conflict of Interest Statement

The author declares that the research was conducted in the absence of any commercial or financial relationships that could be construed as a potential conflict of interest.
